# Improving heart failure outcomes with sodium‐glucose cotransporter 2 inhibitors in different patient groups

**DOI:** 10.1111/dom.15171

**Published:** 2023-06-19

**Authors:** Pardeep S. Jhund

**Affiliations:** ^1^ BHF Glasgow Cardiovascular Research Centre, School of Cardiovascular and Metabolic Health University of Glasgow Glasgow UK

**Keywords:** cardiovascular disease, drug development, drug mechanism, heart failure, SGLT‐2 inhibitor

## Abstract

Sodium‐glucose cotransporter 2 inhibitors (SGLT‐2is) were originally developed for the treatment of hyperglycaemia in type 2 diabetes. Because of regulatory requirements to show the safety of this new class of drugs, a large randomized cardiovascular (CV) outcomes trial was completed but this showed that instead of having a neutral effect on heart failure (HF) outcomes, that these drugs could reduce HF outcomes in this population. Subsequent trials with SGLT‐2is have shown that HF hospitalizations are reduced by 30% and CV death or HF hospitalization by 21% in patients with type 2 diabetes. These findings have extended to patients with HF with reduced and mildly reduced or preserved ejection fraction in whom further HF hospitalizations are reduced by 28% and CV death or HF hospitalizations reduced by 23%, and that it is becoming a central therapy for the treatment of HF. Moreover, the benefit in patients with HF is observed regardless of the presence or absence of type 2 diabetes. Similarly, in patients with chronic kidney disease and albuminuria, with and without type 2 diabetes, the benefit of SGLT‐2is is clearly seen with a 44% reduction in HF hospitalization and 25% reduction in CV death or HF hospitalization. These trials support the use of SGLT‐2is in improving HF outcomes in a broad range of patients, from those with type 2 diabetes, chronic kidney disease and those with pre‐existing HF regardless of ejection fraction.

## 1 INTRODUCTION

**FIGURE 1:**
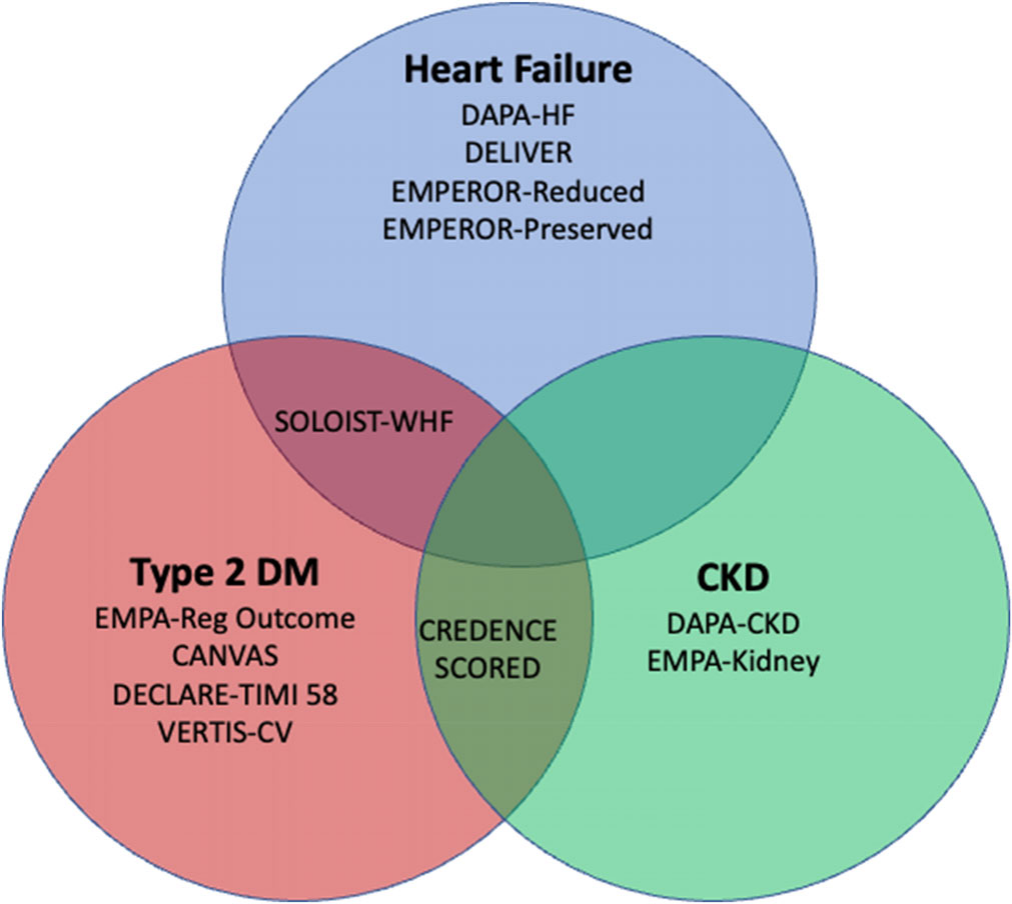
Large prospective randomized trials of sodium‐glucose cotransporter 2 inhibitors in patients with heart failure, type 2 DM and CKD according to the major enrolment criteria. CKD, chronic kidney disease; DM, diabetes mellitus.

The sodium‐glucose cotransporter 2 inhibitors (SGLT‐2is) have been well studied in patients with a wide range of cardiovascular (CV) and metabolic conditions.^1^ This has meant that there are many different groups who now benefit from an SGLT‐2i. One of the most striking features of this class of drugs was the effect that they had on the development of heart failure (HF) in patients with type 2 diabetes, the group in whom they were first tested. This was one of the most impressive findings of the first trial of the SGLT‐2i (empagliflozin) in patients with type 2 diabetes in the EMPA‐Reg Outcome trial.^2^ In that trial, as well as reducing the primary endpoint of CV death, myocardial infarction and stroke, there was a 35% reduction in risk of HF hospitalization. This was seen in all patients and was consistent across many different subgroups. The results of this trial were soon replicated in other trials in patients with type 2 diabetes using other SGLT‐2is.^3^, ^4^, ^5^, ^6^ Throughout each trial there was a consistent effect on HF. Trials were then also conducted in patients with HF^7^, ^8^, ^9^, ^10^, ^11^ and chronic kidney disease (CKD),^12^, ^13^ including those without type 2 diabetes and yet again consistent reductions in HF outcomes were observed. There is overlap between these diseases in their aetiology (e.g. hypertension or atherosclerotic disease both can cause HF and CKD) and because they are aetiologies for each other (e.g. HF can cause type 2 diabetes and type 2 diabetes can cause HF) a consistent benefit in these groups is potentially expected. This review will examine the evidence from the large scale, prospective randomized trials that have been conducted in patients with type 2 diabetes, HF and CKD, that have reported the effect of SGLT‐2is on the prevention and treatment of HF (Figure 1).

## 2 TYPE 2 DIABETES

**FIGURE 2:**
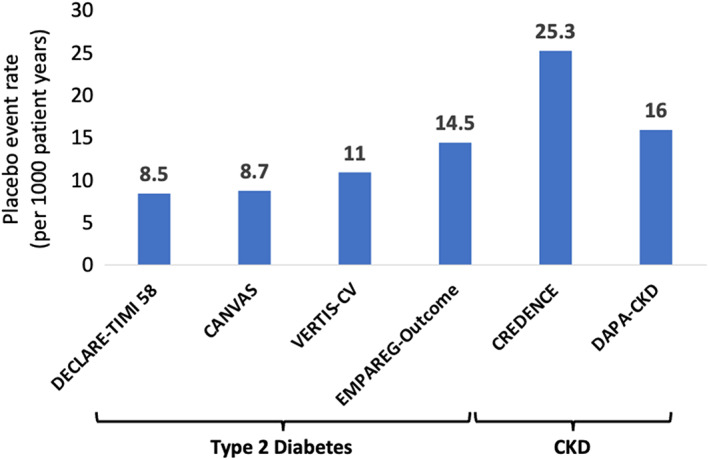
Rate of heart failure hospitalization in trials of sodium‐glucose cotransporter 2 inhibitors in patients with type 2 diabetes or CKD. CKD, chronic kidney disease.

**FIGURE 3:**
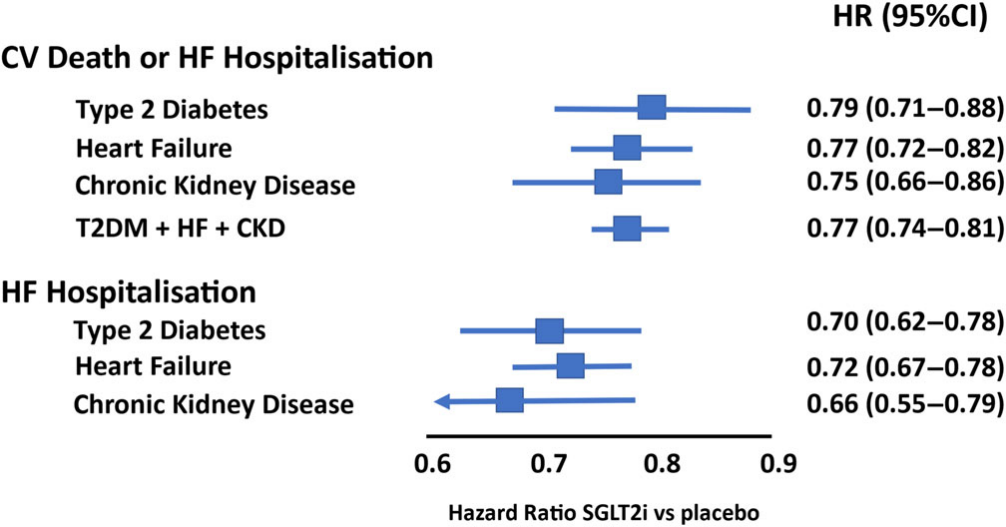
Estimate of treatment effect of sodium‐glucose cotransporter 2 inhibitors (SGLT‐2is) versus placebo on cardiovascular (CV) death or heart failure (HF) hospitalization or HF hospitalization from published meta‐analyses of trials according to major enrolment populations. Estimates for type 2 diabetes (T2DM) taken from Bhatia et al.^23^ for heart failure from Vaduganathan et al.^39^ for chronic kidney disease (CKD) from Qui et al.^43^ and for T2DM and HF and CKD from Baigent et al.^1^ HR, hazard ratio.

A number of trials have examined the effect of SGLT‐2is in patients with type 2 diabetes.^2^, ^3^, ^4^, ^5^, ^6^ The trials were initially designed to examine the safety of these drugs and to show that there was no excess risk of CV events with this class following the requirements of regulatory authorities for new glucose‐lowering therapies. The reduction in CV events that were reported by the first of these trials, the EMPA‐Reg Outcome^2^ was surprising given that previous trials of glucose‐lowering therapies for type 2 diabetes had not shown any significant reduction in CV events.^14^ The trial reported a 14% relative risk reduction in the primary composite of CV death, myocardial infarction or stroke over a median of 3.1 years in patients with type 2 diabetes and established CV disease. In the 7020 patients randomized, there was a significant reduction in the risk of HF hospitalization of 35% [hazard ratio (HR) 0.65; 95% confidence interval (CI) (0.5‐0.85)].^15^ This was an impressive relative risk reduction and when accounting for the risk of CV death in a composite outcome of time to first HF hospitalization or CV death, the relative risk reduction was very similar at HR 0.66 (95% CI 0.55‐0.79). The results were also notable for two other reasons that spurned further research into the use of SGLT‐2is in other patient groups. The first was the size of the relative risk reduction. This was large, and given the risk of developing HF in patients with type 2 diabetes, this was a potentially important step forward in the prevention of CV disease in this high‐risk group. It was known that patients with type 2 diabetes were at high risk of developing HF^16^ and once a patient with diabetes develops HF their risk of death increases.^17^, ^18^ The second reason was that the results brought HF prevention to the fore of the minds of physicians treating patients with type 2 diabetes. Although it was known that HF was common in type 2 diabetes, it was not a common endpoint in clinical trials and it even tended to be relegated to a secondary outcome by regulators.^19^ The results of EMPA‐Reg Outcome led to much speculation and research into the potential mechanism by which this risk reduction may have occurred.^20^, ^21^ However, the primary intended use of these drugs was as a glucose‐lowering therapy for patients with type 2 diabetes and the first trials to report their results with this class of drug were in patients with type 2 diabetes. The next trial in patients with type 2 diabetes to report was the CANVAS trial with canagliflozin.^3^ Again, HF was a secondary outcome but was clearly reduced by canagliflozin in this population of 10 142 patients with type 2 diabetes and established CV disease or multiple CV risk factors. The rate of HF events was lower than observed in EMPA‐Reg Outcomes given the lower risk population randomized (Figure 2) but again there was a significant reduction in HF hospitalization [HR 0.67 (95% CI 0.52‐0.87)] and CV death or HF hospitalization [HR 0.78 (95% CI 0.67‐0.91)], with both estimates being in keeping with EMPA‐Reg Outcome.^2^ Any doubts that the results of the EMPA‐Reg Outcome trial were because of chance evaporated with these results. The next two trials to report enrolled similarly higher‐risk patients with established CV disease or CV risk factors. The DECLARE‐TIMI 58 trial in 17 160 patients with type 2 diabetes compared dapagliflozin with placebo.^4^ This trial had dual primary outcomes of CV death, myocardial infarction or ischaemic stroke (which was the original sole outcome) and CV death or hospitalization for HF (which was added as the results of the other trials became available but before any analysis by the data safety monitoring board on the outcome of CV death, myocardial infarction or ischaemic stroke had been performed). As with previous trials, it reached its primary outcome and yet again reported a significant reduction in HF hospitalization [HR 0.73 (95% CI 0.61‐0.88)] and CV death or HF hospitalization [HR 0.83 (95% CI 0.73‐0.95)]. The final trial conducted in patients with type 2 diabetes and established CV disease was the VERTIS‐CV trial with ertugliflozin.^5^ In 8246 patients the risk of HF hospitalization was reduced by 30% [HR 0.70 (95% CI 0.54‐0.90)] and the risk of CV death or HF hospitalization reduced by 12% but this was not statistically significant [HR 0.88 (95% CI 0.75‐1.03)]. Given the number of trials conducted, meta‐analyses have been conducted over the time span of the release of these trials.^1^, ^22^, ^23^ Initial meta‐analyses confirmed the homogeneity of the effect of SGLT‐2is on HF outcomes in patients with type 2 diabetes.^22^ The most recent estimate from an analysis of EMPA‐Reg Outcome, CANVAS, DECLARE‐TIMI 58 and VERTIS‐CV (all of the trials in patients with type 2 diabetes and established CV disease or risk factors) estimates that SGLT‐2is reduce the risk of HF hospitalization by 30% and CV death or HF hospitalization by 21% (Figure 3).^23^

Given the clear and consistent large reduction in the risk of HF in patients with diabetes, this class is now recommended in guidelines to reduce the risk of HF outcomes.^14^ Furthermore, given the results of the trials above, it is unsurprising that efforts were made to test the efficacy of these drugs in treating HF itself. A number of trials in patients with HF were commenced using dapagliflozin and empagliflozin.

## 3 HEART FAILURE

**FIGURE 4:**
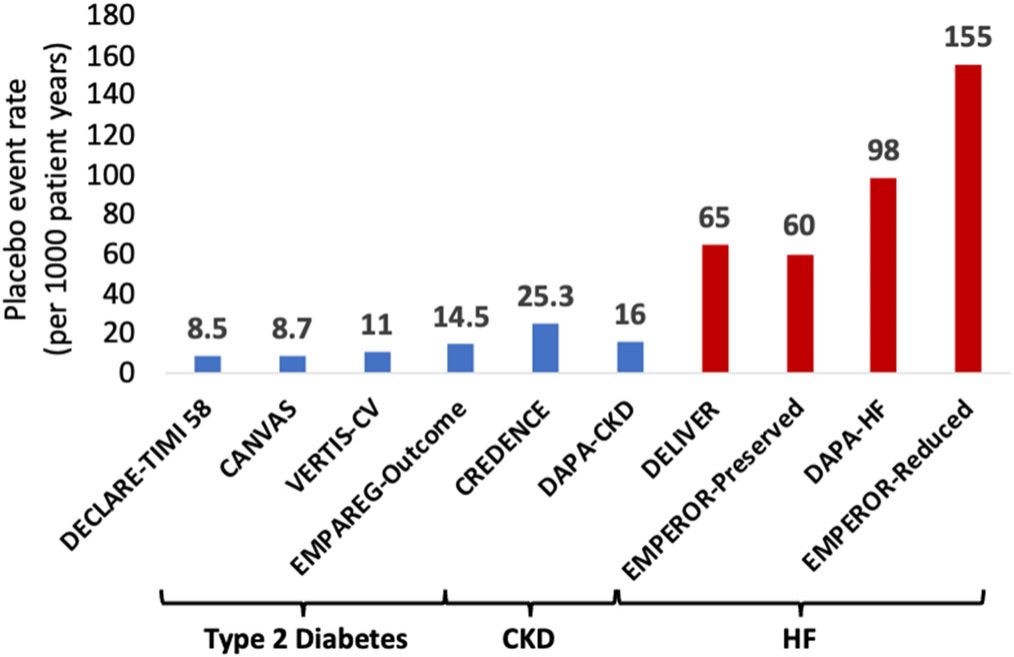
Rate of heart failure (HF) hospitalization in trials of sodium‐glucose cotransporter 2 inhibitors in patients with type 2 diabetes, chronic kidney disease (CKD) or HF. CV, cardiovascular.

With the success of SGLT‐2is in preventing HF outcomes in patients with type 2 diabetes and the common intersection between diabetes and HF, testing a SGLT‐2i as a treatment for HF was a logical next step. However, despite being originally designed as a therapy for blood glucose lowering, the data from the trials in patients with type 2 diabetes along with numerous mechanistic studies suggested glucose independent pathways could be important drivers of their benefit.^21^ The first two trials conducted in HF were both conducted in patients with HF and a reduced ejection fraction. The first of these to report was the DAPA‐HF trial with dapagliflozin in 4744 patients with HF and a left ventricular ejection fraction of ≤40%.^7^ Patients with HF are at much higher risk of further HF events than the patients with type 2 diabetes previously studied in the trials with SGLT‐2is (Figure 4). HF hospitalizations exert a huge burden on health care systems and reducing HF events in this group is a major goal of therapy. Unlike type 2 diabetes where no other therapies had been shown to reduce the risk of HF outcomes,^14^ the SGLT‐2is were tested on top of the guideline recommended therapy for HF, namely angiotensin converting enzyme inhibitors, angiotensin receptor blockers, angiotensin receptor neprilysin inhibitors, beta‐blockers and mineralocorticoid receptor antagonists. DAPA‐HF was powered to examine at the combined outcome of CV death and worsening HF events (HF hospitalizations and urgent HF visits, which comprised of outpatient worsening of HF necessitating the use of intravenous diuretics either in the outpatient department or the emergency room without hospitalization). On breaking down this primary outcome, which was reduced by 26% [HR 0.74 (95% CI 0.65‐0.85)], there was a reduction in the risk of hospitalization for HF [HR 0.70 (95% CI 0.59‐0.83)], total HF hospitalizations and CV death [rate ratio 0.75 (95% CI 0.65‐0.88)], urgent HF visits [HR 0.43 (95% CI 0.20‐0.90)] and CV death [HR 0.82 (95% CI 0.69‐0.98)]. There was also a reduction in all‐cause mortality [HR 0.83 (95% CI 0.71‐0.97)], although because of the hierarchical testing procedure in the trial, this was a statistically nominal reduction. While this was the first time that SGLT‐2is had been tested in HF but also in patients without type 2 diabetes. An important prespecified subgroup analysis of the DAPA‐HF trial therefore was to examine the effect of dapagliflozin in patients with and without type 2 diabetes.^26^ There was a clear reduction in patients with and without type 2 diabetes and without any evidence of heterogeneity of effect, or any statistical interaction in the treatment benefit according to baseline diabetes status, for any of the outcomes examined. The following year the EMPEROR‐Reduced trial with empagliflozin in 3730 patients with HF with reduced ejection fraction confirmed the benefit of SGLT‐2is in HF.^8^ The results of the trial were consistent with the DAPA‐HF trial and a meta‐analysis of the DAPA‐HF and EMPEROR‐Reduced trials confirmed the finding that these drugs reduced HF outcomes and mortality in patients with HF and an ejection fraction ≤40% and that these benefits were observed and consistent in patients in both trials with and without type 2 diabetes.^27^ A final HF outcome that is often overlooked but is an important aim of therapy in HF is improvement in self‐reported health status. Quality of life is low in HF and is consistently a target of therapy yet difficult to improve. The disease‐specific, self‐administered, questionnaire, the Kansas City Cardiomyopathy Questionnaire, was administered in the EMPEROR‐Reduced and DAPA‐HF trials. In both trials, SGLT‐2is improved this key HF outcome with improvements in scores at 8 months compared with placebo.^28^, ^29^ A threshold of a five‐point change is a validated meaningful change and patients who received an SGLT‐2i in these trials were 15%‐20% more likely to experience a five‐point improvement in scores at 8 months and 15% less likely to have a deterioration in their score of ≥5 points at 8 months compared with patients randomized to placebo. Because of the DAPA‐HF and EMPEROR‐Reduced trials, SGLT‐2is are now considered foundational therapy for patients with HF and a reduced ejection fraction.^30^

A second group of patients with HF have proven to be more difficult to treat and are those with an ejection fraction >40% i.e. those with mildly reduced or preserved ejection fraction HF. Previous trials of therapies for HF with reduced ejection fraction such as renin‐angiotensin‐aldosterone system blockers and beta‐blockers were neutral in this population.^30^ A trial with an angiotensin receptor neprilysin inhibitor was also neutral although further analysis suggested a benefit for those with an ejection fraction below normal.^31^ Secondary analysis of trials of drugs in HF with mildly reduced or preserved ejection fraction also suggested that those with HF but an ejection fraction above normal may not benefit from therapies used to treat HF.^32^, ^33^ Therefore, trials of SGLT‐2is in HF with mildly reduced or preserved ejection fraction were eagerly awaited as no previous trials had met their primary endpoint. The first trial of an SGLT‐2i to report was the EMPEROR‐Preserved with empagliflozin, which randomized 5988 patients with HF with mildly reduced or preserved ejection fraction (ejection fraction >40%) to empagliflozin or placebo.^9^ The composite primary outcome was CV death or hospitalization for HF and for the first time a trial in this population reduced its primary endpoint, there was a reduction 21% [HR 0.79 (95% CI 0.69‐0.9)] and this was driven by a reduction in HF hospitalizations [HR 0.71(95% CI 0.61‐0.88)], as the effect on CV death was not significant [HR 0.91(95% CI 0.76‐1.09)]. Nevertheless, a clinically meaningful, statistically significant reduction in HF hospitalizations with empagliflozin made this a landmark trial in the treatment of HF with mildly reduced or preserved ejection fraction. As with the trials of reduced ejection fraction HF there was no evidence of heterogeneity of treatment effect by baseline diabetes status.^34^ As noted above there had been concern that in patients with higher than normal ejection fraction but symptoms of HF that the treatment benefit may be attenuated and in an analysis of the EMPEROR trials across the ejection fraction spectrum, the attenuation of effect appeared to be present although a formal test for interaction was not statistically significant.^35^ To provide a definitive answer to whether SGLT‐2is could improve HF outcomes in those patients with HF and mildly reduced or preserved HF, the results of the DELIVER trial with dapagliflozin were widely anticipated.^10^ The results mirrored those of the EMPEROR‐Preserved trial. There was a 20% reduction in CV death or HF hospitalization [HR 0.80 (95% CI 0.71‐0.91)] and 23% reduction in HF hospitalizations [HR 0.77 (95% CI 0.67‐0.89)] in the 6263 patients randomized.^10^ There were two further analyses of the DELIVER trial that extended the results of the EMPEROR trials. The first was an analysis of the effect of dapagliflozin on HF outcomes across the ejection fraction spectrum. In a pre‐specified analysis, there was no evidence of any heterogeneity of effect across the range of ejection fraction and no suggestion that there was any attenuation of the benefit in this group.^24^ The second important group examined was the 1151 patients (18% of the total trial population) in whom ejection fraction had previously been lower than 40% but had now improved to over 40% by the time of randomization.^36^ This group had previously been excluded for trials in HF with mildly reduced or preserved ejection fraction but in DELIVER they were eligible for randomization. In a pre‐specified analysis of this group the effect of dapagliflozin on a range of HF outcomes was consistent in this group. Therefore, the DELIVER trial also provided evidence for the addition of therapy to this group of patients that had already shown improvement in ejection fraction and that further reductions in HF outcomes were possible with the addition of an SGLT‐2i.

The DAPA‐HF, EMPEROR‐Reduced and EMPEROR‐Preserved trials all included outpatients with HF. Many patients are started on therapy as outpatients because the trials often randomized patients who are in the outpatient setting. During the episode of decompensation clinicians often feel that patients are unstable and therefore may be reluctant to initiate new therapies. This has come about because many of the therapies used for the treatment of HF tend to have adverse effects, which can be difficult to manage when the patient has decompensated, for example low blood pressure or low heart rate and therefore therapies are difficult to use in this population. However, it is known that delaying the use of medications until a patient is discharged results in less use of evidence‐based therapies for HF. Many clinicians aim to initiate therapies while the patient is in hospital thus ensuring that the patient is started on a therapy and increasing the chances that they will continue them, an approach that is endorsed by guidelines.^30^ It was therefore of interest to see whether SGLT‐2is would be beneficial in patients who were experiencing an episode of decompensated HF. There were a number of trials conducted to answer this question. The first of these was in 1222 patients with type 2 diabetes and decompensated HF (irrespective of ejection fraction), i.e. SOLOIST‐WHF.^11^ This trial used the dual SGLT‐2is and SGLT‐1i sotagliflozin and randomized these patients during a hospitalization. Again, in this patient population there was a reduction in the risk of HF hospitalization and the composite of CV death or HF hospitalization, [HR 0.67 (95% CI 0.52‐0.85)]. However, this trial was terminated early because of withdrawal of funding for the trial by the sponsor who was no longer pursuing development of the drug. However, the results of this trial were consistent with other trials in patients with HF and a subgroup of the DELIVER trial. The DELIVER trial also included 654 patients (10% of the trial population) who had been recently admitted with HF and those who were in hospital but no longer receiving intravenous diuretics.^37^ The benefits of dapagliflozin in reducing HF outcomes in this group were again consistent with no evidence that the efficacy of dapagliflozin was reduced in patients with HF who were enrolled during or shortly after a hospitalization. One further trial in this population with HF is notable, the EMPULSE trial with empagliflozin in patients with decompensated HF.^38^ As with SOLOIST‐WHF there was no ejection fraction specified but patients with and without type 2 diabetes were randomized. Only short‐term outcomes were examined in a hierarchical win ratio approach (all‐cause death, number of HF events and time to first HF event, or a ≥5‐point difference in change from baseline in the Kansas City Cardiomyopathy Questionnaire Total Symptom Score at 90 days) but the results were consistent with the other trials of SGLT‐2is in HF [win ratio 1.36 (95% CI 1.09‐1.68)].

A meta‐analysis all of the HF trials showed a clear and significant reduction in the risk of HF outcomes in patients with HF regardless of ejection fraction (Figure 3).^39^ Overall, in patients with HF, the SGLT‐2is reduced HF hospitalizations by 28% and CV death or HF hospitalization by 23%. In this meta‐analysis the effects were consistent across a range of different subgroups as observed in each trial individually.

One final HF outcome that has been analysed in the trials of patients with HF is the progression of kidney disease. Developing CKD and deterioration of kidney function are important outcomes in HF as poorer kidney function limits the use of other drugs used in the treatment of HF. In the trials of SGLT‐2is in type 2 diabetes, the kidney protective effect of these drugs was noted and in HF, SGLT‐2is also slow the rate of decline in estimated glomerular filtration rate (eGFR) that is observed in patients with HF.^25^, ^40^ Just as type 2 diabetes intersects with HF, HF intersects with CKD as it does with type 2 diabetes (Figure 1). Therefore, trials of SGLT‐2is in patients with CKD were also conducted at the same time as many of the trials in patients with HF and type 2 diabetes.

## 4 CHRONIC KIDNEY DISEASE

Patients with CKD are at a high risk of developing HF (Figure 2). HF is an important cause of hospitalization in this population and therefore preventing HF outcomes is an important goal of therapy in patients with CKD. The first SGLT‐2is to be studied in patients with CKD was canagliflozin in the CREDENCE trial.^6^ This trial randomized 4401 patients with CKD (eGFR) of 30 to <90 ml/min/1.73 m^2^ of body surface area and type 2 diabetes who had evidence of albuminuria [ratio of albumin (mg) to creatinine (g), >300 to 5000] and were treated with the renin‐angiotensin system blockade. The aim of the trial was to determine if canagliflozin could reduce the risk of progression of kidney disease in a composite of reaching end‐stage kidney disease, a doubling of serum creatinine, death from kidney or CV causes. The trial met its primary composite endpoint with a 30% reduction in risk [HR 0.70 (95% CI 0.59‐0.81)] giving for the first time an indication that SGLT‐2is could be used in patients with CKD. In addition to preventing the worsening of kidney disease in the CREDENCE trial, there was a significant reduction in risk of hospitalization for HF of 49% [HR 0.61 (95% CI 0.47‐0.80)] and CV death or HF hospitalization [HR 0.69 (95% CI 0.57‐0.83)]. The SCORED trial in 10 584 patients with sotagliflozin also enrolled patients with type 2 diabetes and eGFR 25‐60 ml/min/1.73 m^2^ and risk factors for CV disease.^41^ Although the primary endpoint was initially a reduction in CV death, myocardial infarction or stroke, this was changed during the course of the trial to CV death or HF hospitalization or urgent HF visit. However, as with the other trial with sotagliflozin, SOLOIST‐WHF, the sponsor withdrew funding, and the trial was terminated early. Despite this there was a reduction in the primary composite outcome of 26% [HR 0.74 (95% CI 0.63‐0.88)]. More recently, there have been two large, randomized trials in patients with CKD with and without diabetes.^12^, ^13^ The first was the DAPA‐CKD trial with dapagliflozin in 4304 patients with an eGFR between 25 and 75 ml/min/1.73 m^2^, and a urinary albumin‐to‐creatinine ratio between 200 and 5000 mg/g.^12^ Dapagliflozin reduced the risk of HF hospitalization by 49% [HR 0.51 (95% CI 0.34‐0.76)] and CV death or HF hospitalization by 29% [HR 0.71 (95% CI 0.55‐0.92)] and the effect did not vary by the presence or absence of HF at baseline.^42^ A meta‐analysis of SCORED and DAPA‐CKD suggested that SGLT‐2is reduce the risk of HF hospitalizations by 44% [HR 0.66 (95% CI 0.55‐0.79)] and CV death or HF hospitalization by 25% [HR 0.75 (95% CI 0.66‐0.86)].^43^ The most recent trial to present the results of the effect of an SGLT‐2i in patients with CKD was the EMPA‐Kidney trial with empagliflozin.^13^ In 6609 patients with an eGFR of 20‐45 ml/min/1.73 m^2^ or eGFR of 45‐90 ml/min/1.73 m^2^ and a urinary albumin‐to‐creatinine ratio of ≥200, empagliflozin reduced the risk of progression of kidney disease or death from CV causes but the reduction in the composite of CV death or HF hospitalization did not reach statistical significance [HR 0.84 (95% CI 0.67‐1.07)]. The most recent comprehensive meta‐analysis of the SGLT‐2i trials has incorporated EMPA‐Kidney with all of the trials of SGLT‐2is in type 2 diabetes, HF and CKD, and estimated the effect on CV death or HF hospitalization to be a reduction of 23% [HR 0.77 (95% CI 0.74‐0.81)].^1^

## 5 MECHANISMS OF ACTION

Despite the remarkable consistency of the benefit of the SGLT‐2is on a range of outcomes in patients with HF, type 2 diabetes and CKD, there is still uncertainty as to how these drugs work in each setting.^20^, ^21^, ^44^, ^45^, ^46^, ^47^, ^48^, ^49^, ^50^, ^51^, ^52^ Despite being developed as glucose‐lowering medications, they are effective in patients without type 2 diabetes with HF and kidney disease. It may be that different postulated mechanisms are more or less important in each disease. Moreover, it may be that certain mechanisms are more important during different phases in a disease. For example, longer term left ventricular remodelling may be important in HF with reduced ejection fraction^53^ but does not explain the benefit in HF with the preserved ejection fraction nor the reduction in events seen in shorter trials with patients with acutely decompensated HF.^38^

## 6 CONCLUSION

In addition to being a major burden in patients with established HF, HF outcomes are common in patients with type 2 diabetes and CKD. The SGLT‐2is have repeatedly shown that they reduce HF outcomes in large, randomized trials and that the degree of reduction is clinically meaningful. SGLT‐2is are central to the management of HF, type 2 diabetes and CKD.

## CONFLICT OF INTEREST STATEMENT

PSJ reports speakers' fees from AstraZeneca, Novartis, Alkem Metabolics, ProAdWise Communications, Sun Pharmaceuticals and Intas Pharma; advisory board fees from AstraZeneca, Boehringer Ingelheim and Novartis; research funding from AstraZeneca, Boehringer Ingelheim, Analog Devices Inc. and Roche Diagnostics. PSJ employer, the University of Glasgow, has been remunerated for clinical trial work from AstraZeneca, Bayer AG, Novartis and Novo Nordisk. Director, Global Clinical Trial Partners (GCTP).

## Data Availability

Data sharing is not applicable to this article as no new data were created or analyzed in this study.
